# 6-Aminonicotinamide enhances the efficacy of 5-aminolevulinic acid-mediated photodynamic therapy for neuroblastoma

**DOI:** 10.1186/s12885-025-15231-4

**Published:** 2025-11-25

**Authors:** Satoshi Muramatsu Okamura, Vipin Shankar Chelakkot, Zayar Linn, Yume Onishi, Kana Nakahata, Hidemi Toyoda, Hiroki Hori

**Affiliations:** 1 Department of Medical Education, Mie University Graduate School of Medicine, 2-174, Edobashi, Tsu, Mie 514-8507 Japan; 2 Department of Cancer Biology, Cleveland Clinic Lerner Research Institute, Cleveland, OH USA; 3 Mie Prefectural General Medical Center, Mie, Japan; 4 Nishinomiya Municipal Central Hospital, Hyogo, Japan; 5 Department of Pediatrics, Mie University Graduate School of Medicine, Mie, Japan; 6 Suzuka University of Medical Science, Mie, Japan

**Keywords:** 6-Aminonicotinamide, Glutathione redox system, Photodynamic therapy, 5-Aminolevulinic acid, Lipid peroxidation, Neuroblastoma

## Abstract

**Background:**

Photodynamic therapy (PDT) utilizing 5-aminolevulinic acid (5-ALA) as a photosensitizing precursor is an approved treatment modality for several types of cancers. However, the increased generation of antioxidants in cancer cells renders them resistant to PDT. MYCN-amplified neuroblastoma shows increased production of reactive oxygen species (ROS) and relies on the glutathione redox system for ROS detoxification. We tested the effectiveness of combining 6-aminonicotinamide (6-AN), a glucose-6-phosphate dehydrogenase inhibitor that modulates nicotinamide adenine dinucleotide phosphate and glutathione redox balance, with PDT for treating neuroblastoma.

**Methods:**

Cellular protoporphyrin IX (PpIX) accumulation, cell proliferation, morphological changes, induction of cell death, redox status, and lipid peroxidation were evaluated in neuroblastoma cells treated with 5-ALA-mediated PDT with or without 6-AN.

**Results:**

6-AN enhanced cytotoxicity of 5-ALA-mediated PDT in MYCN-amplified neuroblastoma cells. The enhanced efficacy was attributed to the increase in intracellular PpIX along with the suppression of the glutathione redox system. An analysis of cell death mechanisms revealed that 5-ALA-mediated PDT combined with 6-AN induced necrosis with lipid peroxidation.

**Conclusion:**

6-AN enhances the cytotoxicity of 5-ALA-mediated PDT and promotes membrane lipid peroxidation in neuroblastoma cells. 5-ALA-mediated PDT combined with 6-AN could be a potential treatment strategy for neuroblastoma.

**Supplementary Information:**

The online version contains supplementary material available at 10.1186/s12885-025-15231-4.

## Background

Photodynamic therapy (PDT), a clinically approved, minimally invasive procedure, is used to treat various cancers, including superficial basal cell carcinoma, esophageal cancer, non-small cell lung cancer, and precancerous conditions such as actinic keratosis [1]. 5-Aminolevulinic acid (5-ALA)-mediated PDT utilizes 5-ALA, which is metabolically converted to protoporphyrin IX (PpIX) via the heme biosynthesis pathway. PpIX acts as a photosensitizer compound in cancer cells. PpIX accumulates selectively in cancer cells due to the inherently lower activity of PpIX-metabolizing enzyme, ferrochelatase (FECH), than in normal cells. Upon light activation, PpIX generates reactive oxygen species (ROS) predominantly in cancer cells, inducing cancer cell-specific cytotoxicity [2].

Photoimmunotherapy, which uses light-activated antibody–drug conjugates to selectively target and kill cancer cells, has recently gained attention [3]. However, the high cost and the process of antigen identification significantly limit its accessibility. In contrast, 5-ALA-mediated PDT represents an affordable, straightforward, and efficacious approach, making it particularly appropriate for cancer treatment in low- and middle-income countries [4].

The World Health Organization's Global Initiative for Childhood Cancer aims to increase global childhood cancer survival rates to 60% by 2030 [5]. The superficially located childhood cancers, such as retinoblastoma and Burkitt's lymphoma of maxillary sinuses, represent favorable candidates for 5-ALA-mediated PDT. Additionally, the approach can also be applied intraoperatively for brain and thoracoabdominal tumors, as it has been for adult cancers. Furthermore, it offers distinct advantages over ionizing radiation in terms of cost, equipment and the potential for repeated application. This treatment can be delivered at a primary care center or even at home with a simple phototherapy device, and integrated into multimodal curative or palliative care approaches at a cancer center.

A significant challenge in anticancer therapy is the emergence of treatment resistance. One key mechanism contributing to the development of treatment resistance involves the upregulation of antioxidant defenses resulting from changes in the redox metabolism [6]. Therapies like PDT, whose anticancer activity depends on the induction of oxidative stress, are susceptible to reduced efficacy under conditions of elevated antioxidant capacity in cancer cells. The pentose phosphate pathway (PPP) and glutathione redox system constitute major cellular antioxidant mechanisms [7]. The PPP, a metabolic branch of glycolysis, is critical for nicotinamide adenine dinucleotide phosphate (NADPH) generation. NADPH is essential for the capacity of the glutathione redox system to scavenge diverse oxidants. With upregulated glycolysis [8], cancer cells activate the PPP to generate high levels of NADPH, reinforcing their antioxidant defense. To address the potential antioxidant-related resistance to PDT, especially in glycolytic cancers, we targeted glucose-6-phosphate dehydrogenase (G6PD), a rate-limiting enzyme in PPP. Screening for G6PD inhibitors led us to select 6-aminonicotinamide (6-AN), which is known to modulate NADPH and glutathione redox metabolisms [9]. Consequently, we proposed the combination of 6-AN and 5-ALA-mediated PDT in this study.

Advanced neuroblastoma, particularly the MYCN-amplified form, which exhibits highly aggressive properties, enhances glycolysis [10] and relies on reduced glutathione (GSH) for ROS detoxification [11, 12]. A previous report showed that high MYCN levels are correlated with the altered expression of proteins involved in multiple metabolic processes, including enhanced glycolysis and increased oxidative phosphorylation [13]. Another article reported that the MYCN-amplification has a major influence on the maintenance of aerobic glycolysis, also known as the Warburg effect [14]. Therefore, upregulated glycolysis in MYCN-amplified neuroblastoma represents a compelling target for this treatment strategy.

In this study, we hypothesized that combining G6PD inhibition with 5-ALA-mediated PDT would overwhelm GSH defenses and amplify the activity of ROS, resulting in the induction of enhanced cytotoxicity against MYCN-amplified neuroblastoma cells. The findings from our investigation revealed that 6-AN modulated the redox system and increased the cytotoxicity of 5-ALA-mediated PDT in MYCN-amplified neuroblastoma cells. Notably, 6-AN increased intracellular PpIX accumulation. These findings strongly suggest that 5-ALA-mediated PDT combined with 6-AN represents a promising therapeutic strategy for overcoming resistance and improving outcomes in aggressive, glycolytic cancers.

## Materials and methods

### Materials

5-Aminolevulinic acid hydrochloride and 6-AN were purchased from Tokyo Chemical Industry Co., Ltd. (Tokyo, Japan). Glutathione peroxidase 4 (GPX4) inhibitor (1S, 3R)-RSL3 and ferroptosis inhibitor liprostatin-1 (Lip-1) were obtained from Cayman Chemical (Ann Arbor, MI, USA).

### Neuroblastoma cells and cell culture

We utilized three human neuroblastoma cell lines (SJ-N-JF, NB-19, and NH-12), whose characteristics were previously described [15–17]. SJ-N-JF and NB-19 were provided by St. Jude’s Children’s Research Hospital (Memphis, TN, USA) and NH-12, by JCRB cell bank (Osaka, Japan). SJ-N-JF and NB-19 exhibit MYCN-amplification [16, 17] while NH-12 is a cell line without MYCN-amplification. All these cell lines were maintained in Roswell Park Memorial Institute 1640 (RPMI-1640) medium (Nakalai Tesque, Kyoto, Japan) supplemented with 10% fetal bovine serum and incubated at 37 °C in a humidified 5% CO_2_ atmosphere.

### PDT

A uniform light field was created with a light-emitting diode (LED) array designed with reference to a previous literature [18]. The array consisted of 150 (10 × 15) red LEDs (OS5RKA3131A, OptoSupply Ltd., Hong Kong, China) surrounded by mirrors on four sides, resulting in an illuminated field of 6.9 × 10.7 cm. The LED peak wavelength was 633 nm, measured by a UV1650 PC spectrophotometer (Shimadzu Corporation, Kyoto, Japan). The irradiance, measured at 11 points with an LP1 laser power meter (Sanwa Electric Instrument Co., Ltd., Tokyo, Japan), reached 17.5 mW/cm^2^ (SD = 0.7).

The cells seeded in 96-well plates or 8-well chambered slides (ibidi, Gräfelfing, Germany) were incubated for 24–36 h. The cells were pretreated with 6-AN or dimethyl sulfoxide (DMSO) (the vehicle control for 6-AN) for 18 h, followed by 5-ALA or DMSO (the vehicle control for 5-ALA) for 6 h. After the treatment, the cells were rinsed twice with Hanks' balanced salt solution (HBSS) (Nakalai Tesque) and then irradiated with light.

### Cytotoxicity assay and combined effect analysis

To estimate the cytotoxicity of 5-ALA-mediated PDT combined with 6-AN, a Tetrazolium-based colorimetric assay was performed 24 h after irradiation using the Cell Counting Kit-8 (CCK-8) (Dojindo, Kumamoto, Japan) following the manufacturer's protocol. The absorbance at 450 nm was read with a Multiskan FC Microplate Photometer (Thermo Fisher Scientific, MA, USA). The percentage of dehydrogenase activity relative to the vehicle control was calculated using the following equation:

$$\begin{aligned} &\%\;control\;of\;dehydrogenase\;activity \\ &=100\;\times\;\frac{(absorbance\;\lbrack treated\rbrack\;-\;absorbance\;\lbrack media\rbrack)}{(absorbance\;\left[vehicle\;control\right]\;-\;absorbance\;\left[media\right])} \end{aligned}$$

Additionally, the combined therapeutic effect of 6-AN and 5-ALA-mediated PDT was quantitatively evaluated using the Chou and Talalay method [19]. The combination index (CI) was calculated using CompuSyn software version 1.0 (ComboSyn, Inc., NJ, USA), with CI < 1 indicating synergism, CI = 1 indicating an additive effect, and CI > 1 indicating antagonism.

### Optical microscopy

All optical images were acquired using a Keyence BZ-X710 microscope (Keyence, Osaka, Japan) equipped with an LED light source (3.7 W) and a 2/3-inch 2.83-million-pixel monochrome CCD camera. An S PlanFluor ELWD ADM 20xC/0.45 NA air immersion objective lens (Nikon, Tokyo, Japan) or a CFI Plan Apo λ 40x/0.95 NA air immersion objective lens (Nikon) was used.

### Terminal deoxynucleotidyl transferase (TdT) dUTP nick-end labeling (TUNEL) assay

A TUNEL assay was performed 24 h after irradiation with a Click-iT TUNEL Alexa Fluor 488 imaging kit (Thermo Fisher Scientific) following the manufacturer's protocol. First, the cells were washed once with Phosphate-Buffered Saline (PBS) (Nakalai Tesque) and then fixed for 15 min at room temperature using 4% paraformaldehyde (Nakalai Tesque) in PBS. After the fixation, the cells were permeabilized for 20 min at room temperature using 0.25% Triton X-100 (Nakalai Tesque) in PBS. After permeabilization, positive control cells were treated with DNase I for 15 min and rinsed once with deionized water. A reaction with TdT was then performed for incorporation of 5-Ethynyl-2'-deoxyuridine 5'-triphosphate (EdUTP) into double-stranded DNA strand breaks. This TdT reaction process consisted of pre-treatment with TdT reaction buffer for 10 min at room temperature and treatment with TdT reaction cocktail for 60 min at 37 °C in a 100% humid atmosphere. The TdT reaction cocktail contained TdT reaction buffer, EdUTP, and TdT recombinant in the proportions specified in the manufacturer's protocol. All reagents were supplied in the imaging kit. Following the TdT reaction, the cells were incubated with the Click-iT reaction cocktail for 30 min at room temperature, protected from light. This step utilized a copper (I)-catalyzed click reaction between azide and alkyne groups. The Click-iT reaction cocktail containing Alexa Fluor 488 azide was prepared following the manufacturer's protocol. After the Click-iT reaction, the cells were washed with 3% BSA in PBS for 5 min. For staining DNA, the cells were incubated for 15 min at room temperature with Hoechst 33342 solution, protected from light. DNA-stained images were obtained with an OP-87762 filter (Keyence) consisting of a 360/40 nm excitation and a 460/50 nm emission filter set, and a 400 nm dichroic mirror. TdT staining images were obtained with an OP-87763 filter (Keyence) consisting of a 470/40 nm excitation and a 525/50 nm emission filter set, and a 495 nm dichroic mirror.

### Annexin-V-FITC/Propidium Iodide assay

An Annexin-V-FITC/Propidium Iodide (PI) assay was performed 30 min after light irradiation with an Annexin-V-FITC Apoptosis Detection Kit (Nakalai Tesque) following the manufacturer's protocol. First, the cells were washed twice with PBS and immersed in 100 µL Annexin-V binding buffer, and then 5 µL Annexin-V solution and 5 µL PI solution were added. The cells were incubated in the dark at room temperature for 15 min, and then another 400 µL Annexin-V-binding buffer was added. Annexin-V-FITC-stained images were obtained with an OP-87763 filter. PI staining images were obtained with an OP-87764 filter (Keyence) consisting of a 545/25 nm excitation and a 605/70 nm emission filter set, and a 565 nm dichroic mirror.

### NADPH/NADP^+^ quantification

MYCN-amplified SJ-N-JF neuroblastoma cells were seeded at a cell density of 1.5 × 10^4^/cm^2^ in 75 cm^2^ flasks, incubated for 24 h, and then treated with 6-AN for 24 h. After rinsing twice with PBS, the cells were collected in a 1.5 ml microtube, and NADPH and its oxidized form (NADP^+^) were measured with the NADPH/NADP Assay Kit-WST (Dojindo) following the manufacturer's protocol. The absorbance was read at 450 nm with a Multiskan FC Microplate Photometer to determine NADPH and total NADP(H) contents. NADP^+^ levels were calculated by subtracting NADPH from total NADP(H). The results were presented as the NADPH/NADP^+^ ratio.

### GSH/GSSG quantification

MYCN-amplified SJ-N-JF neuroblastoma cells were seeded at a cell density of 1.5 × 10^4^/cm^2^ in 25 cm^2^ flasks, incubated for 24 h, and then treated with 6-AN for 24 h. After rinsing twice with PBS, the cells were harvested in a 1.5 ml microtube, mixed with 80 μL of 10 mM HCl, and lysed by freeze–thaw cycles. After centrifugation at 8000 × *g* for 10 min, the supernatant was supplemented with 20 µL of 5% sulfosalicylic acid and collected in a 1.5 ml microtube. Oxidized glutathione (GSSG, glutathione disulfide) and GSH concentrations in the supernatant were measured with the GSSG/GSH Quantification Kit (Dojindo) according to the manufacturer's instructions. The absorbance was read at 405 nm (Multiskan FC Microplate Photometer) to determine the GSSG and total glutathione concentrations. The GSH concentration was calculated as total glutathione – (GSSG × 2). The results were presented as the GSH/GSSG ratio.

### Cellular PpIX measurement

The cells seeded in 96-well plates were incubated for 24 h. The cells were then pretreated with different concentrations of 6-AN or DMSO (the vehicle control) for 18 h, followed by 5-ALA or DMSO (the vehicle control) for 6 h. The cells were rinsed twice with HBSS and lysed with radioimmunoprecipitation assay (RIPA) buffer. PpIX fluorescence in the cell lysate was measured with a 2030 ARVO X-2 multilabel reader (Perkin Elmer, MA, USA) with a 405-nm excitation filter and a 620-nm emission filter.

### Lipid peroxidation analysis

Following light irradiation, SJ-N-JF cells were incubated with Lipid Peroxidation Probe BDP 581/591 C11 (L267, Dojindo) for 30 min in a 5% CO_2_ atmosphere at 37 °C and rinsed twice with HBSS. Prior to fluorescence microscopic imaging, the cells were incubated for 3 h in HBSS at 37 °C in a humidified 5% CO_2_ atmosphere. Green fluorescence, which is a marker of lipid peroxidation, was detected with an OP-87763 filter (Keyence) consisting of a 470/40 nm excitation and a 525/50 nm emission filter set, and a 495 nm dichroic mirror. Red fluorescence from the unreacted probe was detected with an OP-87765 filter (Keyence) consisting of a 560/40 nm excitation and a 630/75 nm emission filter set, and a 585 nm dichroic mirror. The fluorescence images were obtained with black balance calibration, which enabled us to correct for camera offset and dark current, thereby allowing reliable quantification of fluorescence intensity. The fluorescence intensities were quantified with ImageJ software version 1.54d (National Institutes of Health, Bethesda, MD, USA) [20]. The lipid peroxidation index was calculated as the green/red fluorescence intensity ratio [21]. Additionally, a lipid hydroperoxide imaging was performed. Following light irradiation, SJ-N-JF cells were incubated with 20 µM Liperfluo (L248, Dojindo) for 30 min in a 5% CO_2_ atmosphere at 37 °C and rinsed twice with HBSS. Then, the cells were incubated for 3 h in HBSS at 37 °C in a humidified 5% CO_2_ atmosphere, followed by fluorescence microscopic imaging with an OP-87763 filter.

### Assessment of the ferroptosis pathway

To investigate the role of ferroptosis in the cytotoxic mechanism of 5-ALA-mediated PDT, we utilized a ferroptosis inhibitor, Liproxstatin-1 (Lip-1), and a glutathione peroxidase 4 (GPX4) inhibitor, (1S, 3R)-RSL3. In this experiment, we substituted 6-AN with (1S, 3R)-RSL3 to evaluate the pure effect of lipid peroxide accumulation produced by 5-ALA-PDT. SJ-N-JF cells were pre-treated with (1S, 3R)-RSL3 for 6 h. Then, the cells were treated with a ferroptosis inhibitor Lip-1 or DMSO (the vehicle control for Lip-1) and then treated with 250 µM 5-ALA or DMSO (the vehicle control for 5-ALA) for 6 h. After the treatment, the cells were rinsed twice with HBSS and irradiated for 15 min. CCK-8 assays were performed 24 h after light irradiation.

### Statistical analysis

Statistical analysis was performed with R software version 4.3.2. Group comparisons were performed to assess significant differences with two-tailed unpaired Student's *t* tests, one-way ANOVA, or two-way ANOVA, with Tukey's post hoc test where appropriate. Significance was defined as *P* < *0.05*.

## Results

### PDT-induced cytotoxicity enhanced by 6-AN

**Fig. 1:**
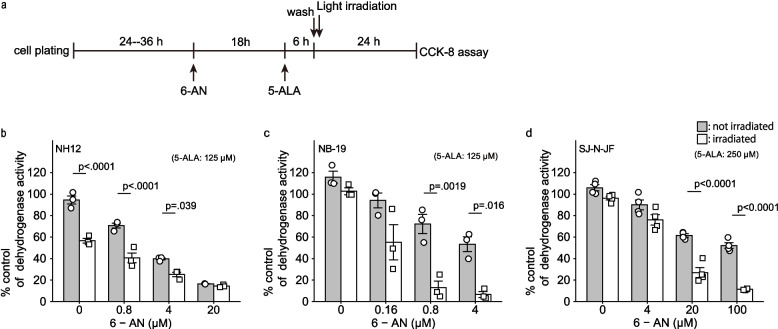
PDT-induced cytotoxicity enhanced by 6-AN. The outline of the experiment is indicated in (**a**). Cells were treated with various concentrations of 6-AN for 18 h, followed by 5-ALA for 6 h and irradiated with light (white column) for 15 min or shielded from light (gray column), and then assayed for the dehydrogenase activity 24 h after irradiation. The dehydrogenase activity of three human neuroblastoma cells treated with PDT with or without 6-AN was measured with CCK-8 assay. The results were shown for each cell line: (**b**) NH-12, (**c**) NB-19 and (**d**) SJ-N-JF. Data are expressed as mean ± SE of % control for dehydrogenase activity, obtained from independent experiments (**b** and** c**, *n* = 3; **d**, *n* = 4). Statistical significance was calculated with two-way ANOVA with Tukey's multiple comparison test

To assess the effect of 6-AN on the cytotoxicity of 5-ALA-mediated PDT, we treated three neuroblastoma cell lines, NH-12, NB-19, and SJ-N-JF, with 6-AN (0—100 µM) followed by 5-ALA-mediated PDT (Fig. 1a). Neither light irradiation nor 5-ALA treatment alone exerted any significant effect on cytotoxicity. NH-12 cells were intrinsically sensitive to 5-ALA-meidated PDT (Fig. 1b), whereas NB-19 and SJ-N-JF cells were resistant. Increasing the concentration of 5-ALA to 250 µM partially overcame the resistance in SJ-N-JF. Crucially, 6-AN significantly enhanced the cytotoxicity of 5-ALA-mediated PDT in the resistant cell lines, with 6-AN concentrations exceeding 0.8 µM for NB-19 and 20 µM for SJ-N-JF (Fig. 1c, d). The two-way ANOVA showed a statistically significant interaction between 6-AN and 5-ALA-mediated PDT (*P* = 0.0001). The CI for the combination of 6-AN and 5-ALA-mediated PDT was less than 1 (Supplementary Fig. S1a-d), suggesting the synergism between 6-AN and 5-ALA-mediated PDT.

### Immunohistochemical and morphological changes in cells treated with 6-AN and PDT

**Fig. 2:**
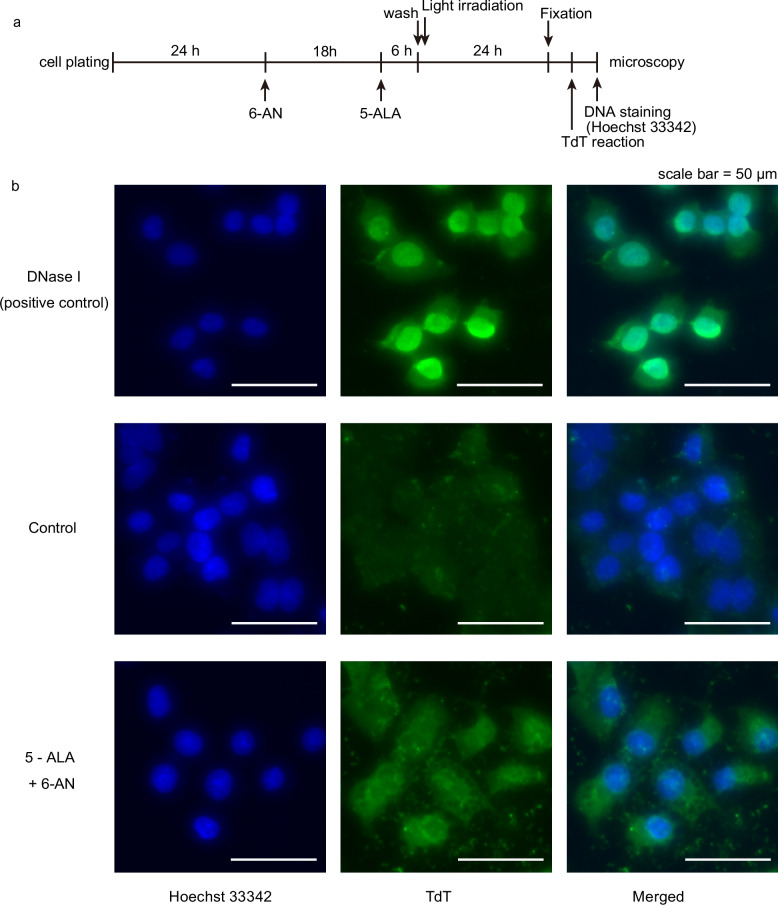
Images of TUNEL assay in SJ-N-JF cells after 6-AN treatment followed by PDT. Cells were treated with 100 µM 6-AN or DMSO, and 500 µM 5-ALA or DMSO, followed by irradiation for 10 min. The outline of the experiment is indicated in (**a**). The pictures are representative fluorescence microscopy images of SJ-N-JF cells stained with Hoechst 33342 (blue) and TdT (green) (**b**). Images were obtained with an S Plan Fluor ELWD ADM 20xC objective lens. The acquisition settings were as follows: exposure time, 0.83 s; gain, + 6 dB; excitation intensity, 100%; transmitted light intensity, 0%; aperture stop, 0% (fully open). Brightness and contrast were adjusted equally across samples using ImageJ. No other image manipulations were performed. A scale bar indicates 50 µm

To elucidate the mechanisms of cell death induced by 5-ALA-mediated PDT combined with 6-AN, we examined immunohistochemical and morphological changes following treatment in SJ-N-JF cells, which are MYCN-amplified and the most resistant to PDT. TUNEL-positive cells were not observed 24 h after the treatment with 100 µM 6-AN and 500 µM 5-ALA (Fig. 2a, b). Annexin-V-FITC/PI assay 30 min after the treatment (100 µM 6-AN and 500 µM 5-ALA) revealed that the majority of cells were Annexin-V-negative/PI-positive, with a small number of scattered Annexin-V-positive/PI-positive cells. The finding suggested that necrosis was the main cause of cell death. Annexin-V-positive/PI-positive cells were presumed to be cells with membrane rupture in which cell membrane-derived phosphatidylserine was stained with Annexin-V (Supplementary Fig. S2a, b). Cells positive for Annexin-V and negative for PI, indicating early phase apoptosis, were not observed.

**Fig. 3:**
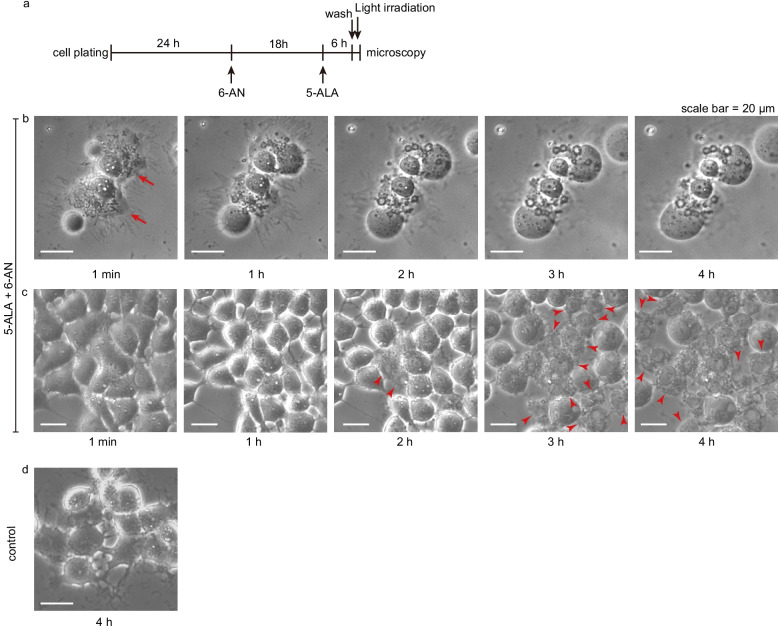
Sequential changes of the cell membrane after 6-AN treatment followed by 5-ALA-mediated PDT. The outline of the experiment is indicated in (**a**). The pictures are representative phase contrast microscopy images of the treated cells at different time points from 1 min post-irradiation. Ruptured (red arrows in **b**) and unruptured cells (**c**) were observed. The ruptured cells displayed ballooning before disruption, with neighboring cells exhibiting similar changes over time (red arrowheads in **c**). The control cells maintained the cell structures 4 h after irradiation (**d**). Images were obtained with an S Plan Fluor ELWD ADM 20xC objective lens. The acquisition settings were as follows: exposure time, 1/15 s; gain, + 6 dB; excitation intensity, 25%; transmitted light intensity, 0%; aperture stop, 0% (fully open)

A morphological analysis 1 min after the treatment (Fig. 3a-c) revealed a mixture of ruptured and intact cells. The affected cells formed vesicle-like structures that gradually ballooned with time (Fig. 3b). Interestingly, the neighboring cells maintaining the cellular integrity subsequently displayed vesicle formation and ballooning, similarly to that observed in the affected cells (Fig. 3c). The control cells maintained their integrity (Fig. 3d) during the period between 1 min and 4 h after light irradiation. These sequential changes (shown in Fig. 3) suggested that necrosis was the major mechanism of cell death. Crucially, the hallmarks of apoptosis, such as apoptotic bodies, were not observed. From these findings, it was speculated that necrotic cell death might be initiated by lipid peroxidation, leading to subsequent membrane permeabilization.

### Changes in the glutathione redox balance, accumulation of PpIX and lipid peroxidation in cells treated with 6-AN and PDT

**Fig. 4:**
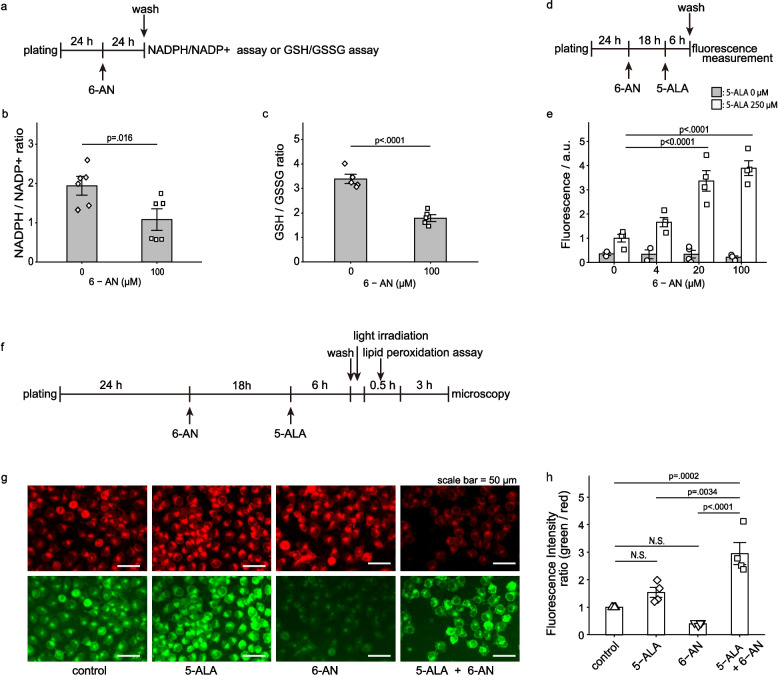
Redox balance, PpIX accumulation and lipid peroxidation after the treatment. The outline of the experiments for NADPH/NADP^+^ or GSH/GSSG quantification is indicated in (**a**). SJ-N-JF cells were treated with or without 6-AN (0 or 100 µM) for 24 h. Fold changes in the NADPH/NADP^+^ ratio (**b**) and the GSH/GSSG ratio (**c**) are indicated. Data are presented as mean ± SE. The number of samples measured in independent experiments was *n* = 6 in (**b**) and *n* = 5 in (**c**). Statistical significance was calculated with two-tailed unpaired Student's *t*-test. The outline of the experiment for cellular PpIX fluorescence measurement is indicated in (**d**). Cells were treated with 6-AN (0 to 100 µM) for 18 h, followed by 5-ALA (0 or 250 µM) for 6 h. Fold change in cellular PpIX fluorescence is indicated in (**e**). Data are presented as mean ± SE, obtained from independent experiments (*n* = 4). Statistical significance was calculated using two-way ANOVA and Tukey's multiple comparison test. The outline of the experiment for lipid peroxidation analysis is indicated in (**f**). Cells were treated with 6-AN (0 or 100 µM), followed by 5-ALA (0 or 500 µM), and then irradiated for 10 min. The pictures are representative fluorescence microscopy images showing reduced (red) and oxidized (green) probe fluorescence (**g**). Images were obtained 3.5 h after irradiation with the S PlanFluor ELWD ADM 20xC objective lens. The acquisition settings were as follows: exposure time, (red) 0.033 s, (green) 10 s; gain, + 6 dB; excitation intensity, 100%; transmitted light intensity, 0%; aperture stop, 0% (fully open). Brightness and contrast settings were applied equally to all samples using ImageJ. No other image manipulation was performed. Lipid peroxidation was measured by quantifying red and green fluorescence from the raw images and is presented as mean ± SE of the fold change compared with controls (**h**). The data were obtained from independent experiments (*n* = 4). Statistical significance was calculated using two-way ANOVA and Tukey's multiple comparison test

To gain further insight into the effect of 6-AN on the cytotoxicity of 5-ALA-mediated PDT, we examined the glutathione redox status and cellular accumulation of PpIX. Cell viability determined with the trypan blue assay before each experiment was greater than 85%, indicating that the assays were performed with viable cells. 6-AN significantly reduced both NADPH (Fig. 4a, b) and GSH (Fig. 4a, c). Furthermore, 6-AN increased cellular PpIX accumulation (Fig. 4d, e). Lipid peroxidation was assessed 3.5 h after light irradiation. The cells with lipid peroxidation were observed only in the combined treatment of 6-AN followed by 5-ALA-mediated PDT (Fig. 4f-h).

Applying the Bliss independence model to the results of lipid peroxidation (Fig. 4h), the predicted effect of the combination therapy, E_P_, was calculated using the following formula:

$${\mathrm E}_{\mathrm P}={\mathrm E}_{5-\mathrm{ALA}}+{\mathrm E}_{6-\mathrm{AN}}-{\mathrm E}_{5-\mathrm{ALA}}\times{\mathrm E}_{6-\mathrm{AN}}$$

where E_5-ALA_ is the experimental effect of 5-ALA alone (= 1.53, presented in Fig. 4h), and E_6-AN_ is the experimental effect of 6-AN alone (= 0.40, presented in Fig. 4h). The experimental effect of the combination therapy (= 2.95, presented in Fig. 4h) was significantly larger than the predicted effect E_P_ (= 1.31, calculated by the formula). Furthermore, Liperfluo staining images obtained 3.5 h after light irradiation depicted the distribution of lipid hydroperoxides within membrane structures (Supplementary Fig. S3a, b). These results indicated the synergistic effect in lipid peroxidation between 6-AN and 5-ALA-mediated PDT.

### Involvement of GPX4 in lipid peroxidation by PDT

**Fig. 5:**
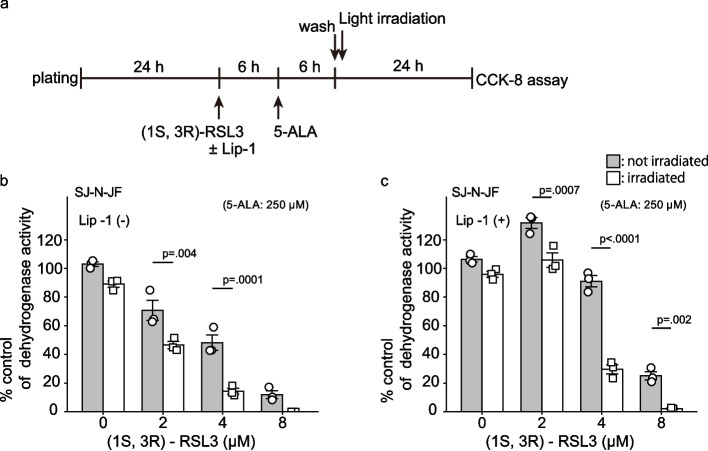
Enhancement of PDT-induced cytotoxicity by GPX4 inhibition. An outline of the experiment is shown in (**a**). SJ-N-JF cells were treated without (**b**) or with (**c**) a ferroptosis inhibitor, Lip-1 (4 µM) and a glutathione peroxidase 4 (GPX4) inhibitor, (1S, 3R)-RSL3 (0 to 8 µM), for 6 h, followed by 250 µM 5-ALA for 6 h and then irradiated for 15 min or shielded from light. The dehydrogenase activity was measured with CCK-8 assay 24 h after irradiation. Data are expressed as mean ± SE of % control for the dehydrogenase activity. The data were obtained from independent experiments (*n* = 3). Statistical significance was calculated using two-way ANOVA with Tukey's multiple comparison test

Initial mechanistic studies revealed that apoptosis was not the major driver of cell death following 5-ALA-mediated PDT and 6-AN combination therapy. Instead, we observed that cell death was consistently preceded by a significant increase in lipid peroxidation. This finding led us to hypothesize cell death related to ferroptosis, a distinct form of regulated cell death defined by iron-dependent lipid peroxidation [22]. Therefore, we conducted a ferroptosis assay to confirm the cytotoxic mechanism. Lip-1 is reported to inhibit ferroptosis through inactivation of lysosomal iron triggering ferroptosis in cancer cells [23], while (1S, 3R)-RSL3 enhances intracellular accumulation of lipid hydroperoxides by inhibiting GPX4. GPX4 is a lipid-repair enzyme that catalyzes the reduction of lipid hydroperoxides into lipid alcohols [24]. Therefore, the enhancement of lipid peroxidation by (1S, 3R)-RSL3 is not limited to the cellular event in ferroptosis. First, we confirmed that GPX4 inhibition by (1S, 3R)-RSL3 enhanced 5-ALA-mediated PDT cytotoxicity similarly to the inhibition by 6-AN (Fig. 5a, b). Second, we examined the cytotoxicity of 5ALA-mediated PDT combined with (1S, 3R)-RSL3 in the condition where ferroptosis was inhibited by Lip-1 (Fig. 5c). Irrespective of the concentration of (1S, 3R)-RSL3, Lip-1 failed to abrogate the cytotoxicity of the treatment, suggesting a ferroptosis-independent mechanism for the cell death that occurred subsequently to lipid peroxidation.

## Discussion

**Fig. 6:**
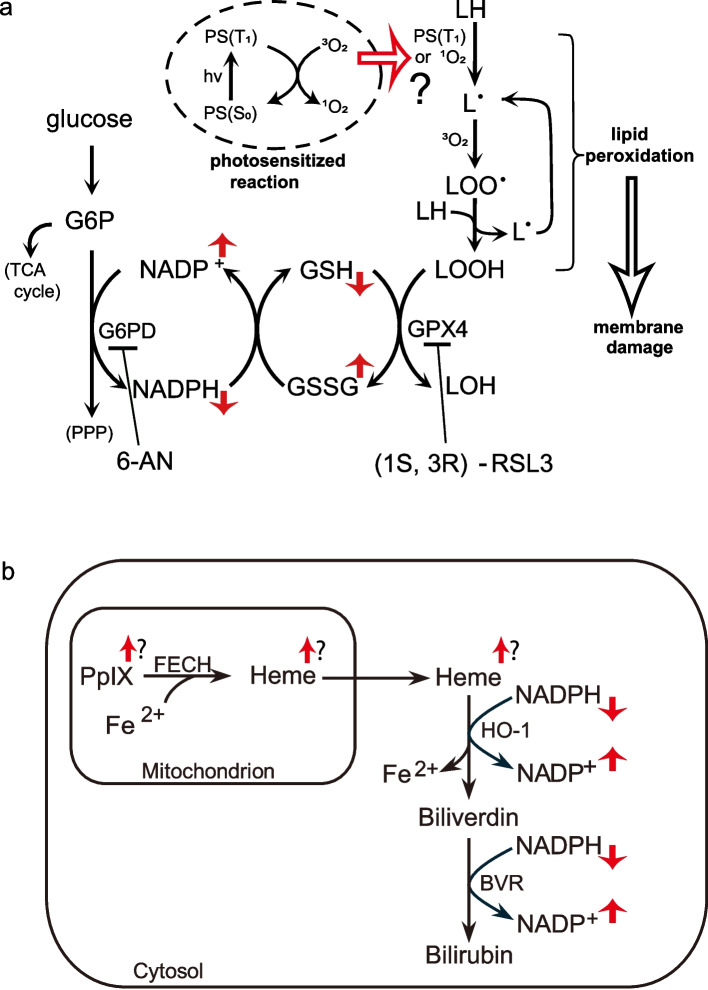
Changes in metabolisms linked to the treatment of 6-AN followed by PDT. Metabolic changes in the redox system and the process of lipid peroxidation induced by 6-AN treatment combined with 5-ALA-mediated PDT are shown in (**a**). Changes in Heme metabolism induced by the treatment are depicted in (**b**). G6P, glucose-6-phosphate; TCA cycle, tricarboxylic acid cycle; PS (S_0_) and PS (T_1_), ground and excited triplet states of photosensitizer; ^3^O_2_ and ^1^O_2_, ground and excited singlet states of oxygen; LH, non-oxidized lipid; L^•^ and LOO^•^, lipid carbon-centered and peroxyl radicals; LOOH, lipid hydroperoxide; LOH, lipid alcohol. FECH, ferrochelatase; HO-1, heme oxygenase 1; BVR, biliverdin reductase

This study demonstrated that inhibiting G6PD with 6-AN enhanced the cytotoxic effect of 5-ALA-mediated PDT against MYCN-amplified neuroblastoma cells, a subtype often resistant to the standard treatments. This enhancement effect was accompanied by necrotic cell death following lipid peroxidation and disruption of the biomembranes. These cellular changes were associated with suppression of the glutathione redox balance and increased intracellular PpIX accumulation caused by 6-AN. Importantly, while many studies focused on approaches to increase ROS production through photosensitizer modifications [25–27], our novel approach adopted G6PD inhibition by 6-AN to lower the potential of antioxidant reduction. NADPH and GSH depletion caused by the inhibition of G6PD could eventually reduce the protective activity of GPX4 in lipid peroxidation (Fig. 6a). Concurrent with the effect on the redox balance, 6-AN increased the accumulation of PpIX in neuroblastoma cells. This effect could be attributed to the role of NADPH in the heme metabolism (Fig. 6b). Heme, which is synthesized from PpIX and Fe^2+^ in the mitochondria, is ultimately catabolized to biliverdin in the cytoplasm through NADPH- and oxygen-dependent processes [28]. Although the mechanism of PpIX accumulation by 6-AN was not fully elucidated in this study, we speculated that G6PD inhibition by 6-AN triggered NADPH depletion, which might inhibit heme degradation and promote the accumulation of heme and PpIX [29, 30]. Further studies using agents such as HO-1 inhibitors, which regulate heme biosynthesis without altering NADPH, should be planned to precisely elucidate this mechanism.

The results from the present study proposed a novel mechanism of PDT-induced cell death in neuroblastoma cells. Our results demonstrated that PDT induced necrotic cell death, characterized by the cell-to-cell spread of membrane disruption originating from lipid peroxidation in the affected cells. Furthermore, we confirmed a synergistic effect between 6-AN and 5-ALA-mediated PDT on both lipid peroxidation and cytotoxicity. Lipid peroxidation is a radical chain reaction consisting of three steps: initiation, propagation, and termination, leading to cell membrane modifications, such as pore formation or permeability changes, resulting in cell death [31]. These morphological changes were observed in neuroblastoma cells treated with 6-AN followed by PDT. Furthermore, we showed that the lipid hydroperoxides (LOOHs) were localized within the membrane structures. In glioma, a neurogenic cancer, PpIX was reported to be localized in the cell membrane [32]. Although we did not examine the localization of PpIX in the cell membrane, PpIX could diffuse into the cell membrane similarly in neuroblastoma cells in the initial process of lipid peroxidation in the cell membrane. Furthermore, since PpIX was reported to be a substrate for efflux pumps of the membrane [33], it could be speculated that PpIX might accumulate in the cytoplasm around the cell membrane. Although the necrotic cell death associated with lipid peroxidation was observed in the combined treatment, apoptosis has conventionally been reported as the primary mechanism of cell death in 5-ALA-mediated PDT [34, 35]. We assumed that the rapid onset of necrosis mediated by lipid peroxidation may have precluded the effective observation of apoptosis induction in this treatment. Based on this speculation, we supposed that treatment with 6-AN followed by 5-ALA-mediated PDT could be effective for apoptosis-resistant cancers [36]. Moreover, we must also consider ferroptosis as a lipid peroxidation-mediated cell death pathway [37, 38]. However, our results showed that a ferroptosis inhibitor, Lip-1, failed to mitigate the cytotoxic effects of PDT enhanced by (1S, 3R)-RSL3. This suggested that PDT with 6-AN could induce lipid peroxidation-mediated cell death in neuroblastoma cells through an alternative pathway. Additionally, it has been reported that G6PD inhibition mitigated ferroptosis [39]. We therefore need further investigations to fully elucidate the molecular mechanism of lipid peroxidation-mediated cell death induced by the combined treatment.

We observed a distinct heterogeneity in cellular response, characterized by the coexistence of cells that retained the cellular structures and those in which membranes were ruptured. This differential response to PDT might be attributable to several biological and physical factors, including differences in light penetration into cells influenced by the cell stratification, variations in the metabolic state associated with the cell cycle, and differences in the distance of cell-to-cell contact. Since light penetration is essential for the practical application of the treatment, we designed the experiments to directly compare a light-irradiated group with a light-shielded control group, thereby validating the photo-activation requirement. Notably, the results demonstrated that the effective concentration of 6-AN was cell-type dependent. SJ-N-JF cells required a considerably higher concentration of 6-AN to exert the cytotoxic effect compared to NB-19 cells, which reportedly share the same MYCN-amplification status. The difference in the effective concentration of 6-AN between the two cell lines may be attributed to the differences in drug uptake and efflux, and metabolisms of antioxidants and protoporphyrin. Future studies focusing on such differences are required to delineate the precise mechanisms underlying the cytotoxic effects of the treatment.

5-ALA-mediated PDT offers inherent advantages in terms of safety, cost-effectiveness, and ease of administration since 5-ALA is an endogenous compound that is already clinically used for cancer imaging. In addition, oral formulations are available. Combining 5-ALA-mediated PDT with 6-AN is expected to exert preferential toxicity toward cancer cells because of their heightened redox activity compared with that of normal cells. While 6-AN transiently suppresses the PPP even in normal cells, PPP function can be readily restored with nicotinamide administration to mitigate potential adverse effects [40]. This treatment strategy targets intrinsic cancer cell vulnerabilities, particularly redox metabolism. In this regard, p53-mutant cancers, where elevated G6PD activity and NADPH production drive hyperactivity of the redox system [41], could be good candidates for this treatment. Although we did not evaluate G6PD gene expression in MYCN-amplified neuroblastoma cells, it was reported that MYCN levels were correlated with altered expression of proteins involved in enhanced glycolysis and increased oxidative phosphorylation [13]. In recent years, studies on G6PD-target therapy have been steadily progressing [42–45]. Our treatment strategy combining 6-AN with 5-ALA-mediated PDT may potentially expand the clinical application of G6PD-targeted therapies. Furthermore, a recent study indicated that *MSI2*, a gene related to the malignant property of neuroblastoma cells, upregulated G6PD and enhanced PPP [46]. Therefore, Neuroblastoma with *MSI2* gene alterations could also be a good candidate for 5-ALA-mediated PDT combined with 6-AN.

Future studies should investigate tumor-microenvironment interactions, particularly considering the impact of lipid peroxides on both tumor and immune cells [47, 48]. For clinical translation, we have to understand the process of lipid peroxidation within these complex environments [49], particularly with respect to the relationship between regulatory T-cell function and lipid metabolism [50]. Furthermore, the role of hypoxia in tumor tissues, which can both reduce PpIX accumulation and enhance the redox system [51], should be investigated. The strategy to enhance light delivery to deeper-seated tumors should also be developed [52, 53].

## Conclusions

The present study provides evidence that combining 6-AN with 5-ALA-mediated PDT effectively induces cell death in MYCN-amplified neuroblastoma cells by disrupting redox balance, increasing cellular PpIX accumulation, and subsequent lipid peroxidation of the biomembranes. While further preclinical validation is necessary before the clinical application, the findings in this study suggested that 5-ALA-mediated PDT potentiated by 6-AN, could be a promising new therapeutic modality for high-risk neuroblastoma.

## Supplementary Information

Supplementary Material 1.

Supplementary Material 2.

Supplementary Material 3.

## Data Availability

The data used and/or analyzed during the current study are available from the corresponding author upon reasonable request.

## Abbreviations

5-ALA: 5-Aminolevulinic acid
6-AN: 6-Aminonicotinamide
DMSO: Dimethyl sulfoxide
G6PD: Glucose-6-phosphate dehydrogenase
GPX4: Glutathione peroxidase 4
GSH: Glutathione
GSSG: Glutathione disulfide (oxidized glutathione)
HBSS: Hanks' balanced salt solution
Lip-1: Liprostatin-1
NADPH: Nicotinamide adenine dinucleotide phosphate
NADP^+^: Oxidized form of nicotinamide adenine dinucleotide phosphate
PpIX: Protoporphyrin IX
PPP: Pentose phosphate pathway
ROS: Reactive oxygen species
TUNEL: Terminal deoxynucleotidyl transferase dUTP nick-end labeling
