# Supplementary material for: 6-Aminonicotinamide enhances the efficacy of 5-aminolevulinic acid-mediated photodynamic therapy for neuroblastoma
Source: BMC Cancer. 2025 Nov 25;25:1815. doi: 10.1186/s12885-025-15231-4 (PMC12648907; doi:10.1186/s12885-025-15231-4)
Supplement: Supplementary file 1 — Supplementary Material 1. [file 12885_2025_15231_MOESM1_ESM.pdf]

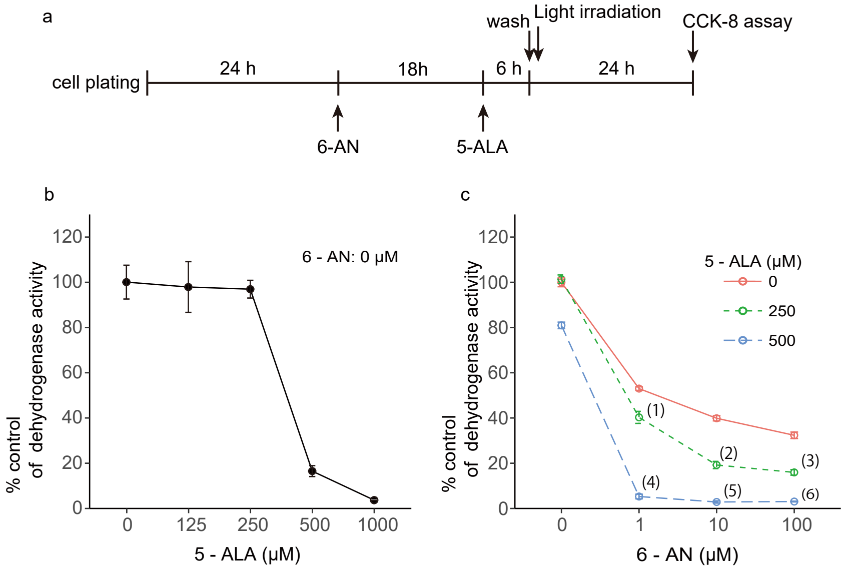

d

CI Data for combination

	5-ALA (μM)	6-AN (μM)	% control	CI
(1)	250.0	1.0	0.403	0.5862
(2)	250.0	10.0	0.192	0.4045
(3)	250.0	100.0	0.159	0.3893
(4)	500.0	1.0	0.0528	0.5766
(5)	500.0	10.0	0.0287	0.4986
(6)	500.0	100.0	0.0306	0.5062

% control = %control of dehydrogenase activity
CI = Combination Index

Fig. S1. CCK-8 assay for analysis of interaction between 5-ALA-mediated PDT and 6-AN

The outline of the experiment is shown in (**a**). The line graph on the left shows the representative cytotoxicity of 5-ALA-mediated PDT against SJ-N-JF cells (**b**). The line graph on the right shows representative cytotoxicity of 6-AN against SJ-N-JF cells with or without 5-ALA-mediated PDT (**c**). Data are expressed as mean ± SD of % control of dehydrogenase activity from the experiments performed in triplicate. The results of the Chou and Talalay analysis for the interaction between 5-ALA-mediated PDT and 6-AN are indicated in (**d**). The table shows the combination indices (CI) for the combinations indicated by the numbers in parentheses (**c**). “CI < 1” means synergistic.
