# Supplementary material for: 6-Aminonicotinamide enhances the efficacy of 5-aminolevulinic acid-mediated photodynamic therapy for neuroblastoma
Source: BMC Cancer. 2025 Nov 25;25:1815. doi: 10.1186/s12885-025-15231-4 (PMC12648907; doi:10.1186/s12885-025-15231-4)
Supplement: Supplementary file 2 — Supplementary Material 2. [file 12885_2025_15231_MOESM2_ESM.pdf]

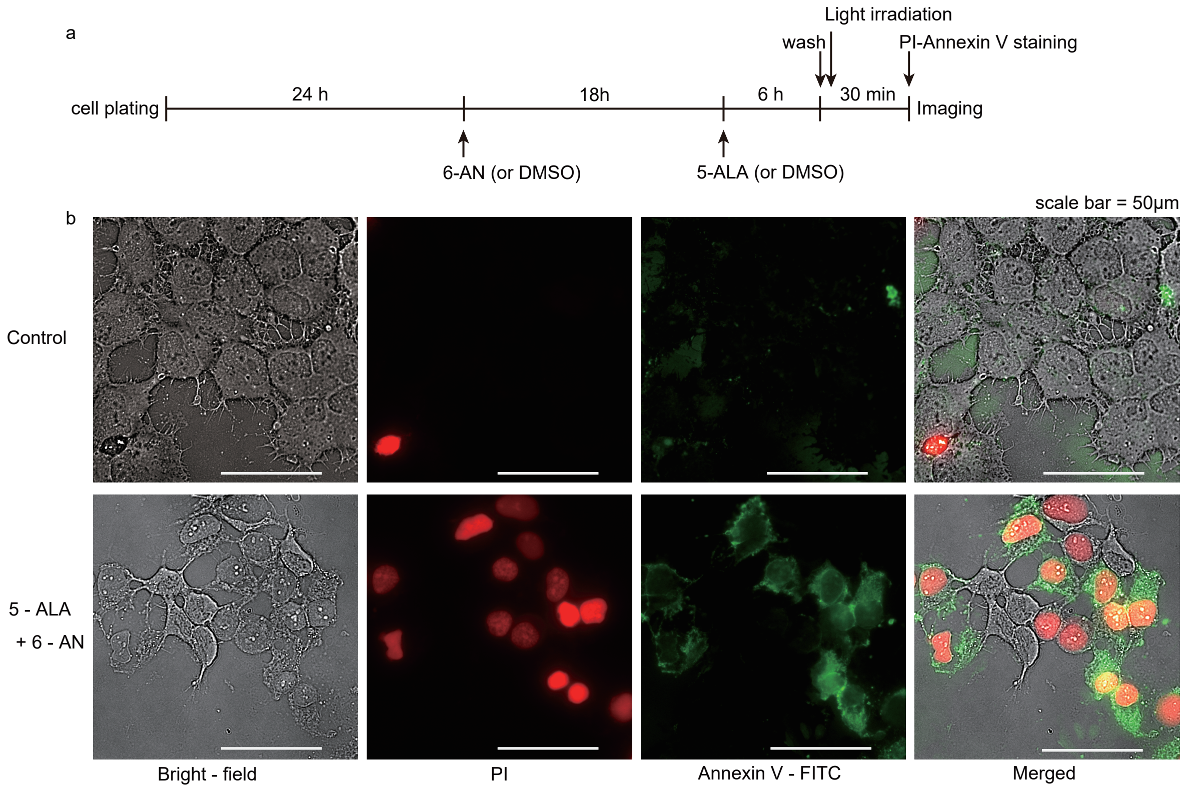

Fig. S2. Images of Annexin V/PI assay in SJ-N-JF cells treated with 6-AN combined 5-ALA-mediated PDT.

The outline of the experiment is shown in (**a**). The pictures are representative images of bright-field microscopy and fluorescence microscopy, and merged images (green, Annexin V -FITC; red, PI). The cells were treated with 100 μM 6-AN or DMSO and 500 μM 5-ALA or DMSO, and then irradiated for 10 min. Images were obtained with a CFI PlanApo λ 40x objective lens. The acquisition settings of fluorescence microscopy were as follows: exposure time, (Annexin V -FITC) 3.5 s, (PI) 0.1 s; gain, +6 dB; excitation intensity, 40%; transmitted light intensity, 0%; aperture stop, 0% (fully open). Brightness and contrast settings were applied equally to all samples using ImageJ before the figure was prepared. No other image manipulation was performed. Scale bar = 50 μm.
