# Supplementary material for: 6-Aminonicotinamide enhances the efficacy of 5-aminolevulinic acid-mediated photodynamic therapy for neuroblastoma
Source: BMC Cancer. 2025 Nov 25;25:1815. doi: 10.1186/s12885-025-15231-4 (PMC12648907; doi:10.1186/s12885-025-15231-4)
Supplement: Supplementary file 3 — Supplementary Material 3. [file 12885_2025_15231_MOESM3_ESM.pdf]

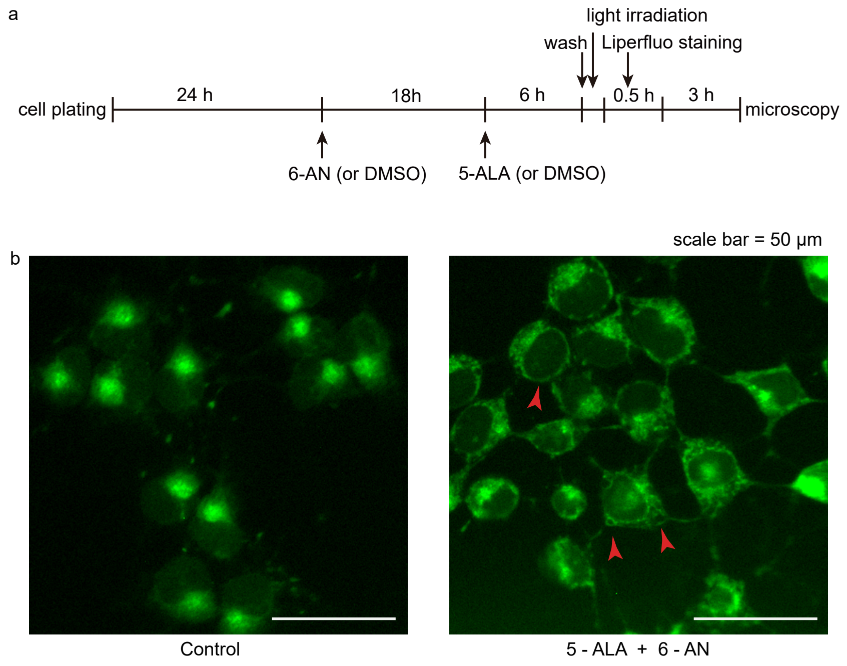

Fig. S3. Fluorescence images of lipid peroxide in SJ-N-JF cells after 6-AN combined PDT.

The outline of the experiment is shown in (**a**). The pictures are representative fluorescence microscopic images showing SJ-N-JF cells stained with Liperfluo **(b)**. Fluorescent membrane structures were observed (red arrows in **b, right**). The cells were treated with 100 µM 6-AN or DMSO and 500 µM 5-ALA or DMSO, and then irradiated for 10 min. Images were obtained 3.5 h after irradiation using an S PlanFluor ELWD ADM 20xC objective lens. The acquisition settings were as follows: exposure time, 28 s; gain, +6 dB; excitation intensity, 100%; transmitted light intensity, 0%; aperture stop, 0% (fully open). Brightness and contrast settings were applied equally to all samples using ImageJ before the figure was prepared. No other image manipulation was performed. Scale bar = 50 µm.
